# P-1754. Reduced Risk of Acute Kidney Injury with Montelukast in Vancomycin-Treated Patients: A Propensity-Matched Cohort Study

**DOI:** 10.1093/ofid/ofaf695.1925

**Published:** 2026-01-11

**Authors:** Ashveen Bains, Paddy Ssentongo, Siddartha Guru, Cory M Hale, Silvana Ribeiro Papp, Chen Song, Zinaida Perciuleac

**Affiliations:** Milton S. Medical Center, Hershey, PA; Penn State Health Milton S. Hershey Medical Center, Hershey, PA; Penn State Health Milton S. Hershey Medical Center, Hershey, PA; Penn State Health Milton S. Hershey Medical Center, Hershey, PA; UPMC, Dover, PA; Penn State Health Milton S. Hershey Medical Center, Hershey, PA; Penn State Health Milton S. Hershey Medical Center, Hershey, PA

## Abstract

**Background:**

Vancomycin is a cornerstone of treatment for serious Gram-positive infections but is associated with nephrotoxicity. Montelukast, a leukotriene receptor antagonist with anti-inflammatory properties, may confer renal protection. We evaluated whether the addition of montelukast to vancomycin is associated with a reduced incidence of acute kidney injury (AKI).

**Figure 1.**
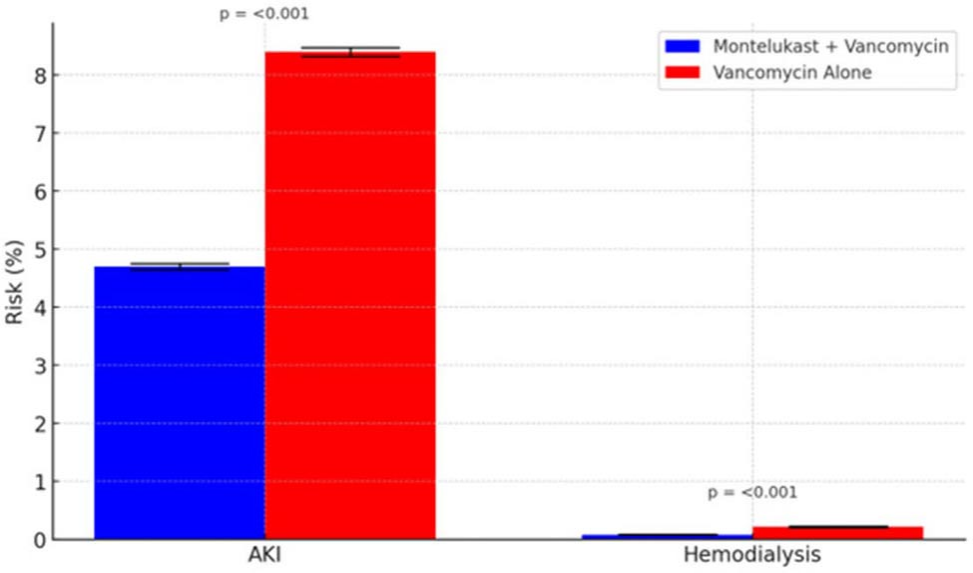
Risk of Acute Kidney Injury and Hemodialysis by Treatment Group.

Bar plots display the 90-day incidence of acute kidney injury (AKI) and receipt of hemodialysis among propensity score–matched patients treated with vancomycin alone versus vancomycin plus montelukast. Error bars represent the standard error of the proportion. The montelukast group had significantly lower rates of both AKI (4.7% vs. 8.4%) and hemodialysis (0.08% vs. 0.22%) compared to vancomycin alone (p < 0.001 for both comparisons).

**Figure:**
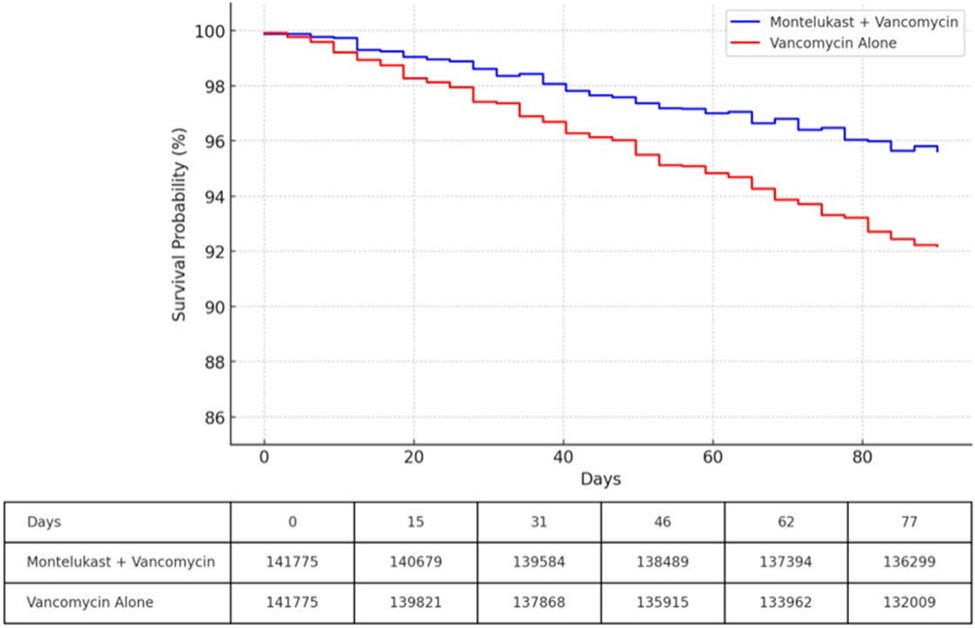

**Methods:**

We conducted a retrospective cohort study using the TriNetX Global Collaborative Network, including adults aged ≥18 years who received vancomycin without preexisting chronic kidney disease. Patients who were on montelukast and received vancomycin initiation formed the intervention group (n=141,775), while those who received vancomycin alone comprised the control group (n=141,775) after 1:1 propensity score matching. Propensity scores were generated using age at index, sex, and race. The primary outcome was incident AKI within 90 days. Secondary outcomes included ICU admission, mortality, and need for hemodialysis. Risks were compared using risk ratios and Kaplan–Meier survival analysis.

**Results:**

Among 283,550 propensity-matched patients (mean age 55.8 years, 62.5% women, 71.1% White), those receiving montelukast and vancomycin had a significantly lower incidence of acute kidney injury (AKI) within 90 days compared to vancomycin alone (4.7% vs. 8.4%; risk ratio [RR], 0.56; 95% CI, 0.54–0.58; *p*< 0.001 Figure 1). The risk of hemodialysis was also reduced (0.08% vs. 0.22%; RR, 0.34; 95% CI, 0.28–0.43; *p*< 0.001). ICU admission occurred less frequently in the montelukast group (0.02% vs. 0.21%; RR, 0.09; 95% CI, 0.06–0.14; *p*< 0.001). Mortality at 90 days was significantly lower with montelukast (4.5% vs. 8.0%; hazard ratio, 0.52; 95% CI, 0.51–0.54; *p*< 0.001, Figure 2). Risks of depression, rash, thrombocytopenia, and hepatitis were not significantly different between groups.

**Conclusion:**

In this large, matched cohort study, the use of montelukast in patients receiving vancomycin was associated with a significantly lower risk of AKI and hemodialysis. The risk for ICU admission and mortality was also lower among patients receiving concomitant Montelukast and vancomycin. These findings suggest a potential nephroprotective role for montelukast that merits further prospective investigation.

**Disclosures:**

All Authors: No reported disclosures

